# Current antimicrobial prescription at outpatient dentistry centers and clinics in tertiary-care hospitals in Tokyo, Japan: A multicenter cross-sectional study

**DOI:** 10.1017/ash.2021.229

**Published:** 2021-12-13

**Authors:** Yasuaki Tagashira, Masao Horiuchi, Atsushi Kosaka, Takuya Washino, Mikihiro Horiuchi, Shutaro Murakami, Itoe Tagashira, Hitoshi Honda

**Affiliations:** 1 Division of Infectious Diseases, Tokyo Metropolitan Tama Medical Center, Tokyo, Japan; 2 Division of Infectious Diseases, Tokyo Medical and Dental University, Tokyo, Japan; 3 Department of Infection Prevention and Control, Tokyo Metropolitan Cancer and Infectious Diseases Center, Komagome Hospital, Tokyo, Japan; 4 Department of Infectious Diseases, Tokyo Metropolitan Bokutoh Hospital, Tokyo, Japan; 5 Department of Pharmacy, Tokyo Metropolitan Hiroo Hospital, Tokyo, Japan; 6 Department of pharmacy, Tokyo Metropolitan Tama Medical Center, Tokyo, Japan; 7 Department of Dysphagia Rehabilitation, Tokyo Medical and Dental University, Tokyo, Japan

## Abstract

**Background:**

Antimicrobial administration is necessary before specific dental procedures to prevent postprocedural infections and complications and antimicrobials are sometimes indicated for the treatment of odontogenic infections. However, antimicrobials are commonly misused by dentists.

**Methods:**

This cross-sectional study was conducted at 4 public, tertiary-care hospitals in Tokyo, Japan, from June to July 2019. We included patients who received an antimicrobial prescription at the outpatient dentistry center or clinic at each participating hospital. The indications for antimicrobial prescription were (1) antimicrobial prescriptions for prophylaxis (APPs) or (2) antimicrobial prescriptions for treatment (APTs). Prescribing patterns were described in terms of antimicrobial choice, timing, and dosages for APPs and APTs.

**Results:**

During the study period, 1,772 patients received an antimicrobial prescription. Among them 1,439 (81.2%) were APPs and 333 (18.8%) were APTs. The most common aim of APP was to prevent local infections and complications following tooth extraction (n = 1,244, 86.4%). The proportion of appropriate APPs was only 0.8% (12 of 1,439). Among 1,439 total APPs, 171 (11.9%) were unnecessary, 32 (2.2%) were inappropriate, and 1,224 (85.1%) were suboptimal. Whereas 101 (30.3%) of 333 APTs were appropriate, the remaining 97 instances (29.1%) were unnecessary, 86 (26.7%) were inappropriate, and 46 (13.8%) were suboptimal.

**Conclusion:**

Inappropriate antimicrobial prescriptions were common among dentists in Japan. Understanding the differences in the current antimicrobial prescribing patterns for prophylaxis and treatment is critically important for implementing an effective antimicrobial stewardship program in dentistry.

The antimicrobial stewardship program (ASP) is a core strategy for optimizing antimicrobial use to prevent the further development of antimicrobial resistance.^1^ Antimicrobial prescriptions by dentists account for 8%–10% of overall antimicrobial prescriptions in high-income countries, including Japan.^2,3^ The World Dental Federation (FDI) has emphasized that antimicrobial agents should be used only when absolutely necessary.^4^ However, ∼80% of antimicrobials prescribed in the dental setting qualify as instances of misuse.^5–7^ A point-prevalence study in the United States also revealed that only 8% of antimicrobials prescribed by dentists were appropriate for the dental setting.^6^ Although the current antimicrobial prescription practices in dentistry are widely known, very few studies have attempted to analyze them using comprehensive clinical data. In the present study, we explored patterns of antimicrobial prescription in the outpatient dentistry centers and clinics at 4 tertiary-care hospitals in Tokyo, Japan.

## Material and methods

### Study design and setting

The present, descriptive, multicenter cross-sectional study was conducted at several public tertiary-care hospitals in the Tokyo Metropolitan Hospital system, including Tokyo Metropolitan Tama Medical Center, Tokyo Metropolitan Bokutoh hospital, Tokyo Metropolitan Cancer and Infectious Diseases Center Komagome Hospital, and Tokyo Metropolitan Hiroo Hospital. All of the participating Tokyo metropolitan hospitals are government-owned tertiary-care centers with a department of dentistry and oral-maxillofacial surgery that also provide outpatient dental care. Data on prescriptions from June 2019 to July 2019 were obtained from the hospital records. The pre-existing ASPs at the participating hospitals consisted of a prospective audit and feedback for intravenous antimicrobial agents used in the inpatient setting. However, there was no outpatient ASP prior to the present study at any of the centers except Tokyo Metropolitan Tama Medical Center, which had a multifaceted ASP in the emergency department.^8^

### Participant selection

All patients visiting the outpatient dentistry center or clinic at each participating hospital during the study period were initially enrolled, then eligible patients who received a prescription for oral antimicrobials at discharge were identified. The antimicrobial prescriptions were classified as (1) antimicrobial prescriptions for prophylaxis (APPs) or (2) antimicrobial prescriptions for treatment (APTs). Whereas APPs were defined as antimicrobial prescriptions for dental procedures in the absence of odontogenic infections, APTs were defined as antimicrobial prescriptions for the treatment of odontogenic infections. For patients with multiple antimicrobial prescriptions during the study period, only the first instance was included for analysis. We applied the following exclusion criteria: (1) age <18 years, (2) no history of oral antimicrobial prescriptions, (3) hospitalization on the same day as the clinic visit, and (4) an oral antimicrobial prescription unrelated to a dental procedure or odontogenic infection.

### Data collection

A list of potentially eligible patients and their basic demographic information were obtained from the hospital records. After excluding patients meeting the exclusion criteria, the following data on the participants were collected by reviewing their electronic medical records (EMR): detailed demographic characteristics, clinical data, dental procedures, clinical diagnosis, indications for antimicrobial prescriptions determined by dentists, and antimicrobial prescription data, including timing, dosage, and intervals. An antimicrobial prescription in the absence of a definitive diagnosis by the treating dentist was considered an instance of just-in-case antimicrobial use. Prescriber information, including postgraduate year (PGY) level, specialization, and board certification, was also tracked.

### Definition of appropriate APP

Antimicrobial prophylaxis against infective endocarditis was considered appropriate if patients with the medical conditions outlined by the American Heart Association guidelines (eg, history of infective endocarditis, presence of a prosthetic valve/material, etc) underwent a procedure involving manipulation of gingival tissue or treatment for periapical lesions or oral mucosa perforation.^9^ Antimicrobial prophylaxis was considered appropriate for tooth extraction and implant placement regardless of any underlying cardiac condition or the presence of a prosthetic joint.^10,11^ Only preprocedural administration was considered appropriate timing for antimicrobial prophylaxis. Antimicrobial prescriptions for other dental procedures, including those not requiring prophylaxis for infective endocarditis, were considered unnecessary. Antimicrobial prophylaxis against prosthetic joint infections was also considered unnecessary (in the absence of indications).^12,13^ Supplementary Table 1 shows the details of appropriate APPs, including indications for procedures and antimicrobial regimens based on current evidence.

### Definition of appropriate APT

The definition of appropriate APTs was based on the clinical guidelines of the Scottish Dental Clinical Effectiveness Programme (SDCEP) and the Faculty of General Dental Practice (FGDP), which list the indications for antimicrobial administration and give recommendations on the type of antimicrobial agent to use.^14^ These guidelines recommend antimicrobial treatment in cases of odontogenic infection with abscess formation and evidence of infection spread (eg, facial swelling, erythematous lesions, and lymphadenitis due to an odontogenic infection) or systemic involvement (fever and malaise). Other indications for appropriate antimicrobial treatment include necrotizing ulcerative gingivitis and pericoronitis with systemic involvement or persistent swelling despite local treatment.^14^ Antimicrobials were also considered appropriate for treating sinusitis in the presence of severe or persistent symptoms with or without purulent discharge lasting at least 7 days^14,15^ A list of recommendations in international infectious disease guidelines and a textbook (Supplementary Table 2)^14,16^ for the treatment of infectious diseases commonly encountered in dentistry was used in the present study to determine the indications for treatment.

### Evaluation of APP and APT appropriateness

Antimicrobial misuse was defined as any antimicrobial prescription failing to meet the criteria in Supplementary Tables 1 and 2. Misuse of antimicrobial prescriptions was further classified as unnecessary, inappropriate, or suboptimal use based on the previously mentioned criteria.^17,18^ Unnecessary use was defined as the use of an antimicrobial agent in patients with no indications, procedures with no indications, a noninfectious condition, and nonbacterial or self-limiting bacterial infections and included antimicrobial use in the context of an uncertain diagnosis. Inappropriate use was defined as the use of an antimicrobial agent not conforming to the current prophylaxis or treatment guidelines. Suboptimal use was defined as the use of an antimicrobial that could have been improved in terms of dosage, timing, or interval. Finally, all discharge antimicrobial prescriptions not meeting the classification of misuse were considered appropriate. Supplementary Figure 2 shows a study flow for the assessment of the necessity and appropriateness of antimicrobial prescriptions.

### Data of interest

The main purpose of the present study was to describe antimicrobial prescribing patterns for prophylaxis and treatment at the outpatient dentistry centers and clinics of the participating hospitals. The institutional review board at each hospital approved this study, and the requirement for patient consent was waived because the study was cross-sectional and did not influence the current management of the enrolled patients.

## Results

Of the 13,980 patients visiting the outpatient dentistry centers and clinics during the study period, 2,380 (17.0 %) received an oral antimicrobial prescription. Of these patients, 608 (4.3%) were excluded (Supplementary Fig. 1), leaving 1,772 patients (12.7%) for analysis. Among the patients receiving an antimicrobial prescription at the outpatient dentistry centers or clinics, 1,439 (81.2%) received an APP and 333 (18.8%) received an APT.

### Characteristics of the patients and prescribing dentists

Table 1 summarizes the baseline characteristics of the patients. The median age of the patients was 44 years (range, 18–100 years), and 43.1% were female. The prevalence of an underlying medical condition requiring infective endocarditis prophylaxis was 1.2% (22 of 1,772). In total, 37 dentists prescribed antimicrobials; their median PGY was 18 years (range, 1–37 years). Also, 7 (18.9%) of the dentists were dentists in training (ie, dental residents), 9 (24.3%) were board-certified dentists in oral and maxillofacial surgery (OMS), 11 (29.7%) were board-certified specialists in OMS, and 6 (16.2%) were board-certified advanced specialists in OMS. The remaining 4 dentists had no valid board certification.

**Table 1.** Baseline Characteristics of Patients With an Antimicrobial Prescription From an Outpatient Dentistry Center or Clinic (N = 1,772)

Characteristics	Total (N = 1,772)
**Demographics**	
Age, median y (range)	44 (18–100)
Sex, female, no. (%)	763 (43.1)
Antimicrobial allergy, no. (%)	53 (3.0)
**Comorbidity/past medical history**, no. (%)	
Valvular disease	19 (1.1)
Post prosthetic valve placement	11 (0.6)
History of infective endocarditis	1 (0.06)
Unrepaired cyanotic chronic heart disease	8 (0.5)
Cardiac transplantation recipient	2 (0.1)
Cardiovascular implantable electronic device placement	17 (1.0)
Coronary artery stent placement	32 (1.8)
Total joint replacement	18 (1.0)
Vascular stent placement	10 (0.6)
Diabetes mellitus	113 (6.4)
Chronic liver disease	15 (0.8)
Chronic kidney disease	34 (1.9)
Connective tissue disease	45 (2.5)
Active malignancy	101 (5.7)
Post solid-organ transplantation	1 (0.06)
Post hematopoietic stem cell transplantation	7 (0.4)
HIV	5 (0.3)
Systemic steroid use (≥ 5 mg) in the last 28 days	29 (1.6)
Chemotherapy in the last 28 days	24 (1.4)
Radiation therapy	21 (1.2)

Note. HIV, human immunodeficiency virus.

### APP in the outpatient dentistry centers or clinics

The chief indications for APP were the prevention of a local infection and complications following tooth extraction (n = 1,244, 86.3%), followed by prophylaxis against infective endocarditis (n = 19, 1.3%) (Supplementary Table 3). The most common dental procedures were wisdom tooth extractions (n = 873, 60.7%), followed by other tooth extractions (n = 389, 27.0%) and biopsies (n = 78, 5.4%). Overall, 1,427 (99.8%) of APPs were considered instances of misuse. The most common timing of APP for tooth extraction and implant placement was postprocedural only (n = 569, 44.9%), followed by preprocedural and postprocedural timing (n = 404, 31.9%).

Among instances of APP misuse (n = 1,427), 171 (11.9%) prescriptions were unnecessary, 32 (2.2%) prescriptions were inappropriate, and 1,224 (85.1%) prescriptions were suboptimal. The most common reason for a suboptimal APP was underdosing and suboptimal timing (ie, the antimicrobials were prescribed after the procedure). Table 2 shows the details of the reasons for the unnecessary, inappropriate, and suboptimal APPs. Table 4 shows that a significant variation in the APP patterns was observed at each hospital.

**Table 2.** Details of Appropriateness of APP per Procedure (N = 1,439)

Procedure (N = 1,439)	Misuse (n = 1,427), No. (%)					Appropriate (n = 12), No. (%)
	Suboptimal (n = 1,224)			Inappropriate (n = 32)	Unnecessary (n = 171)	
	Underdosing (n = 280)^a^	Suboptimal timing (n = 2)^b^	Both (n = 942)			
Wisdom tooth extraction (n = 873)	202 (23.1)	2 (0.2)	648 (74.2)	17 (1.9)	0	4 (0.5)
Other tooth extraction (n = 389)	78 (20.1)	0	288 (74.0)	15 (3.9)	0	8 (2.1)
Biopsy (n = 78)	0	0	0	0	78 (100)	0
Tumor/cyst removal (n = 41)	0	0	0	0	41 (100)	0
Other procedure (n = 58)^c^	0	0	6 (10.3)	0	52 (89.7)	0

a Supplementary Table 1 shows appropriate dosing of antimicrobials for APP.

b Optimal timing of APP means that patients received antimicrobials only preprocedurally. Otherwise, APP (antimicrobials given both pre-and postprocedurally, and postprocedurally only) would be considered suboptimal timing.

c Other procedures included: implant placement (n = 5), implant removal (n = 5), suturing (n = 1), root canal treatment (n = 5), necrotic bone removal (n = 4), frenectomy (n = 3), washing (n = 4), incision and drainage (n = 3), scaling (n = 3), curettage (n = 2), fenestration surgery for ranula (n = 2), suture removal (n = 2), pulpectomy (n = 2), osteoplasty (n = 2), oral vitiligo excision (n = 2), occlusal adjustment (n = 1), dental filling (n = 1), caries removal (n = 1), root planning (n = 1), periodontal surgery (n = 1), orthodontic wire placement (n = 1), demucosation (n = 1), epulis removal (n = 1), crown cutting (n = 1), gauze packing (n = 1), cystectomy (n = 1), foreign material removal (n = 2), bone transplantation (n = 1), sialolith removal (n = 1), and gingival retraction (n = 1).

### APT in the outpatient dentistry centers/clinics

Common diagnoses made by the treating dentists that led to APT included mandibular osteomyelitis (n = 83, 24.9%), apical periodontitis (n = 37, 11.1%), pericoronitis (n = 34, 10.2%), and dental abscess formation (n = 32, 9.6%) (Supplementary Table 4). In total, 226 (67.9%) of 333 APTs were considered instances of misuse. Among 333 APTs, 97 (29.1%) prescriptions were unnecessary, 92 (27.6%) were inappropriate, and 37 (11.1%) were suboptimal. In APTs, antimicrobial use in the absence of clinical indications, use of unnecessarily broad antimicrobials, and underdosing were commonly observed. Table 3 shows the detailed reasons for each type of misuse.

**Fig. 1.**
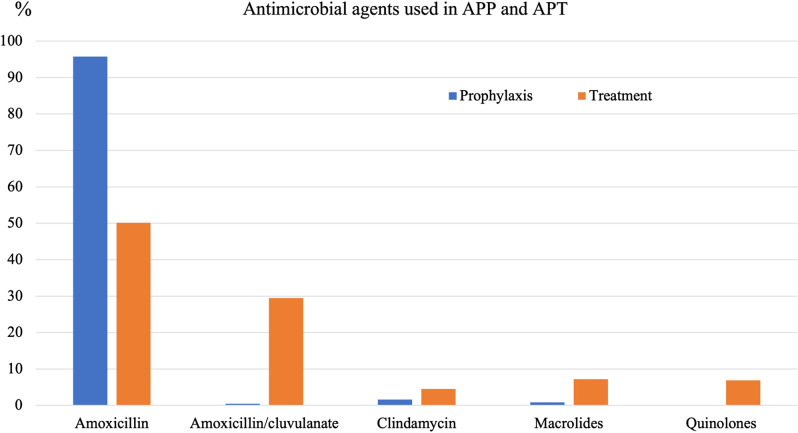
Antimicrobial agents used in APP and APT.

Although amoxicillin and amoxicillin–clavulanate were the 2 most commonly prescribed antimicrobials (n = 265, 79.6%) for APT, non–first-line antimicrobials with broad-spectrum activity, such as macrolides, quinolones, and clindamycin, were also widely prescribed for therapeutic purposes (Fig. 1). As with APPs, a significant variation in the APT pattern was observed at each hospital (Table 4).

**Table 3.** Details of Appropriateness of APT per Physician’s Diagnosis (N = 333)

Diagnosis (N = 333)	Misuse (n = 226), No. (%)			Appropriate (n = 107), No. (%)
	Unnecessary (n = 97)	Inappropriate (n = 92)	Suboptimal (n = 37)	
Mandibular osteomyelitis (n = 83)	0	20 (24.1)	7 (8.4)	56 (67.5)
Apical periodontitis (n = 37)	22 (59.5)	4 (10.8)	10 (27.0)	1 (2.7)
Pericoronitis (n = 34)	16 (47.1)	8 (23.5)	8 (23.5)	2 (5.9)
Dental abscess (n = 32)	10 (31.3)	2 (6.3)	0	20 (62.5)
Facial cellulitis from odontogenic infection (n = 24)	0	17 (70.8)	0	7 (29.2)
Acute odontogenic maxillary sinusitis (n = 24)	0	17 (70.8)	0	7 (29.2)
Acute gingivitis (n = 22)	0	14 (63.6)	2 (9.1)	6 (27.3)
Periodontitis (n = 14)	8 (56.1)	0	6 (42.9)	0
Local infection following post tooth extraction (n = 11)	8 (72.7)	3 (27.3)	0	0
Sialadenitis (n = 6)	0	3 (50.0)	0	3 (50.0)
Osteonecrosis (n = 6)	2 (33.3)	2 (33.3)	1 (16.7)	1 (16.7)
Alveolar osteitis (n = 5)	3 (60.0)	2 (40.0)	0	0
Just in case (n = 7)^a^	7 (100)	0	0	0
Others (n = 28)^b^	21 (75.0)	0	3 (10.7)	4 (14.3)

Note. The percentage (%) in each cell was calculated by the number of antimicrobial uses divided by the number of procedures.

a Antimicrobial prescriptions in the absence of a definitive diagnosis by a dentist was considered to be an instance of just-in-case antimicrobial use.

b Includes dry socket (n = 4), mucositis (n = 4), pericoronitis surrounding implant (n = 3), pulpitis (n = 3), insufficient healing post tooth extraction (n = 2), salivolithiasis (n = 2), oral cyst infection (n = 2), necrotizing ulcerative gingivitis (n = 2), hematoma (n = 1), lymphangitis (n = 1), chronic gingivitis (n = 1), infection caused by impacted tooth (n = 1), animal bite (n = 1), and cheilitis (n = 1).

**Table 4.** Details of Prescribing Patterns Per Hospital

Antimicrobial prescriptions for prophylaxis				
Hospital	Prescribing Pattern for Tooth Extraction or Implant Placement, No. (%)			Prophylactic Antimicrobials in the Absence of Indications, No. (%)
	Preprocedural Only	Pre- and Postprocedural	Postprocedural Only	
A	241/480 (50.2)	36/480 (7.5)	171/480 (35.6)	32/480 (6.7)
B	51/539 (9.5)	351/539 (65.1)	75/539 (13.9)	62/539 (11.5)
C	2/271 (0.7)	15/271 (5.5)	219/271 (80.8)	35/271 (12.9)
D	0/149 (0)	2/149 (1.3)	104/149 (69.8)	43/149 (28.9)

Antimicrobial prescriptions for treatment				
Hospital	Appropriate	Misused		
		Unnecessary	Inappropriate	Suboptimal
A	3/45 (6.7)	20/45 (44.4)	20/45 (44.4)	2/45 (4.4)
B	30/100 (30.0)	26/100 (26.0)	29/100 (29.0)	15/100 (15.0)
C	52/116 (44.8)	22/116 (19.0)	23/116 (19.8)	19/116 (16.4)
D	16/72 (22.2)	29/72 (40.3)	17/72 (23.6)	10/72 (13.9)

## Discussion

This multicenter study revealed that antimicrobials were prescribed in ∼17% of all dental care occasions, with APPs and APTs accounting for 80% and 20% of the prescriptions, respectively.

Antimicrobial misuse was identified in both APPs and APTs. Approximately 10% of APPs were for procedures that did not require antimicrobial prophylaxis. Moreover, even when an APP was indicated, most prescriptions were inconsistent in terms of timing and dosage; only 20% of the patients received a preprocedural APP only, and amoxicillin was rarely prescribed at the standard dosage of 2 g. Although there is no consensus on evidence-based APP in dentistry,^19^ the guidelines of professional societies and a systematic review have suggested that amoxicillin 2–3 g only at the preprocedural timing should be considered the standard regimen^10,11^ and have discouraged postprocedural antimicrobial prophylaxis.^20^ Although the reasons for the inappropriate timing of APPs observed in the present study are unclear, various factors, including the prescribing practices in the workplace, previous education, and inexperience in prophylaxis using higher dosages of amoxicillin at the pre-procedural-only timing, might be associated with inappropriate prescribing behaviors.^21^

Although APT accounted for a relatively small proportion of the total antimicrobial prescriptions, a considerable number of APT (∼30% of the total APT) were considered unnecessary. As noted in the treatment guidelines, odontogenic infections may not always require antimicrobial treatment, and dentists need to determine the need for antimicrobial prescriptions based on clinical data.^14^ Moreover, broad-spectrum antimicrobials, such as macrolides, quinolones, and clindamycin, were prescribed in ∼20% of APT. The misuse of these and other antimicrobials has a number of deleterious effects, including undermining antimicrobial stewardship efforts, promoting antimicrobial resistance, and developing antimicrobial-related adverse drug events.^22^

Another notable finding was evidence of significant variations in APP and APT prescribing practices at the participating institutions. (Supplementary Table 3). The lack of a standardized treatment for odontogenic infections might be contributing to this marked variation in antimicrobial prescribing practices. Moreover, a previous survey suggested that antimicrobial prescribing practices were strongly influenced by the prescribers’ previous education and workplace culture.^21^ Another study also demonstrated that differences in dentists’ level of knowledge about antimicrobial prescribing practices might be contributing to the variations in their prescribing behaviors.^23^ These findings indicate that the current education on antimicrobial stewardship in dentistry is insufficient.^24–27^ For effective antimicrobial stewardship, establishing guidelines for standard APP and APT practices is urgently needed. Educational opportunities for dentists to learn about the indications for antimicrobial prophylaxis per type of dental procedure and for antimicrobial treatment of odontogenic infections is the first necessary step toward establishing antimicrobial stewardship in dentistry.

This study had several limitations. Because of its cross-sectional study design, the findings of the present study may not represent long-term trends in antimicrobial prescribing practices in Japan. Second, the findings may not be generalizable to other healthcare settings, such as private clinics, which account for 80% of dental treatment facilities in Japan.^28^ Moreover, factors associated with the misuse of antimicrobial prescriptions were not identified in this study. Determining the appropriateness of antimicrobial prescriptions through an EMR review might have introduced a bias due to the limited information contained in the records. The duration of antimicrobial prescriptions was not tracked because only the first instance of antimicrobial prescription was considered. The incidence of adverse drug events due to antimicrobial use may also have been underestimated because patients might have visited other institutions after experiencing symptoms.

In conclusion, the 2 categories of antimicrobial prescribing practice in dentistry examined by this study were APP and APT, both of which were common. Although we did not examine the differences between these 2 patterns in detail, the characteristics of misuse occurring in each differ significantly. The main problems in APPs consisted of inappropriate dosing and timing whereas in APTs the main problems were a high proportion of unnecessary antimicrobial prescriptions and the excessive use of broad-spectrum antimicrobials. The lack of well-established evidence in dentistry for prophylactic and therapeutic antimicrobial practices further complicates the situation. Because significant quantities of antimicrobials are prescribed by dentists, optimizing antimicrobial prescribing practices in dentistry is crucial. Our findings suggest that a better approach to using antimicrobials for APPs and APTs is urgently needed.

## Supplementary material

For supplementary material accompanying this paper visit https://doi.org/10.1017/ash.2021.229. click here to view supplementary material
