# Supplementary material for: Current antimicrobial prescription at outpatient dentistry centers and clinics in tertiary-care hospitals in Tokyo, Japan: A multicenter cross-sectional study
Source: Antimicrob Steward Healthc Epidemiol. 2021 Dec 13;1(1):e64. doi: 10.1017/ash.2021.229 (PMC9495602; doi:10.1017/ash.2021.229)
Supplement: Supplementary file 1 [file S2732494X21002291sup001.docx]

**Supplementary title: Current antimicrobial prescription at outpatient dentistry centers/clinics in tertiary care hospitals in Tokyo: a multicenter cross-sectional study**

Authors: Yasuaki Tagashira MD, PhD, Masao Horiuchi MD, Atsushi Kosaka MD, Takuya Washino MD, Mikihiro Horiuchi BS, Shutaro Murakami BS, Itoe Tagashira DDS, PhD, Hitoshi Honda MD, PhD

Supplementary Table 1. Details of appropriate antimicrobial prescription for prophylaxis

| Patient population | Procedure | Drug, Dosage, Timing | References |
| --- | --- | --- | --- |
| Patients with a medical condition listed in the American Heart Association guidelines | Procedure involving manipulation of gingival tissue or treatment for periapical lesions or oral mucosa perforation | Amoxicillin 2g orally pre-procedure only  For patients unable to take oral medication:  ampicillin 2g, cefazolin or ceftriaxone 1g intravenously pre-procedure only  For patients with a history of penicillin or amoxicillin allergy:  Cephalexin 2g or clindamycin 600mg, azithromycin or clarithromycin 500mg orally pre-procedure only  For patients with a history of penicillin or amoxicillin allergy unable to take oral medications:  cefazolin or ceftriaxone 1g or clindamycin 600mg intravenously pre-procedure only | Wilson et al.^1^ |
| Patients with no medical conditions listed in the American Heart Association guidelines | Tooth extraction | Amoxicillin 2g or 3g orally pre-procedure only | Suda et al.^2^ |
| Patients with no medical conditions listed in the American Heart Association guidelines | Implant placement | Amoxicillin 2g or 3g orally pre-procedure only | Esposito et al.^3^ |
| Patients with no medical conditions listed in the American Heart Association guidelines, including patients with a prosthetic joint | Dental procedures except tooth extraction and implant placement | Antimicrobial prophylaxis not recommended | Lockhart et al.^4^  Sollecito et al.^5^ |

Supplementary Table 2. Appropriate regimen for antimicrobial prescription for treatment

| Diagnosis | Drug, Dosage, Timing |
| --- | --- |
| Odontogenic infections, including dental abscess | Amoxicillin 500 mg PO three times daily^6^  Amoxicillin-clavulanate 250 mg PO three times daily^6^  Clindamycin 150 mg PO four times daily^6^  Clarithromycin 250 mg PO two times daily^6^  For patients with a history of penicillin allergy:  Metronidazole 200 mg PO three times daily^6^ |
| Pericoronitis | Amoxicillin 500 mg PO three times daily^6^  For patients with a history of penicillin allergy:  Metronidazole 200 mg PO three times daily^6^ |
| Sinusitis | Amoxicillin 500 mg PO three times daily^6,7^  Amoxicillin-clavulanate 500 mg PO three times daily ^7^  For patients with a history of penicillin allergy:  Doxycycline 100 mg PO twice times daily^6^ |
| Necrotizing ulcerative gingivitis | Amoxicillin 500 mg PO three times daily^6^  Metronidazole 500 mg PO three times daily^7^  For patients with a history of penicillin allergy:  Metronidazole 200 mg PO three times daily^6^ |
| Acute simple gingivitis | Amoxicillin-clavulanate 500 mg PO three times daily^7^  Clindamycin 450 mg PO three times daily^7^ |
| Sialadenitis and suppurative parotitis | Amoxicillin-clavulanate 500 mg PO three times daily^7^  Clindamycin, 600 mg PO three times daily^7^ |
| Periodontitis | Amoxicillin-clavulanate, 500 mg PO three times daily^7^ |
| Mucositis | Oral antimicrobial therapy not recommended.^7^ |
| Endodontic infections | Amoxicillin 500 mg PO three times daily^8^ |
| Acute periodontitis and acute apical abscess | Effects of systemic antimicrobial unknown^9^ |

Supplementary Table 3. Details of antimicrobial prescriptions for prophylaxis (APP) in oral surgery at outpatient dentistry centers/clinics in tertiary care hospitals (N=1,439)

| Characteristics | N=1,439 |
| --- | --- |
| Demographics |  |
| Age, year, median (range) | 41 (18-98) |
| Female sex | 612 (42.5) |
| Antimicrobial allergy | 39 (2.7) |
| Comorbidity/past medical history |  |
| Valvular disease | 11 (0.8) |
| Post prosthetic valve placement | 8 (0.6) |
| History of infective endocarditis | 1 (0.07) |
| Unrepaired cyanotic chronic heart disease | 8 (0.6) |
| Cardiac transplantation recipient | 2 (0.1) |
| Cardiovascular implantable electronic device placement | 11 (0.8) |
| Coronary artery stent placement | 24 (1.7) |
| Total joint replacement | 12 (0.8) |
| Vascular stent placement | 8 (0.6) |
| Allergy to antimicrobial agents | 39 (2.7) |
| Primary dental procedure |  |
| Wisdom tooth extraction | 873 (60.7) |
| Tooth extraction other than wisdom tooth | 389 (27.0) |
| Biopsy | 78 (5.4) |
| Tumor removal | 23 (1.6) |
| Cyst removal | 18 (1.3) |
| Other* | 58 (4.0) |
| Reason for antimicrobial prescriptions for prophylaxis |  |
| Prevention of local infection and complications following tooth extraction | 1, 244 (86.4) |
| Prevention of infective endocarditis | 19 (1.3) |
| Prevention of implant failure | 5 (0.4) |
| Antimicrobial agent prescribed for prophylaxis |  |
| Amoxicillin | 1,378 (95.8) |
| Clindamycin | 23 (1.6) |
| 3rd-generation cephalosporin | 13 (0.9) |
| Clarithromycin | 7 (0.5) |
| Amoxicillin/clavulanate | 7 (0.5) |
| Azithromycin | 5 (0.3) |
| Other** | 4 (0.3) |
| Timing of antimicrobial administration in all instances of prophylactic use |  |
| Post-procedural only | 697 (48.4) |
| Pre- and post-procedural | 433 (30.1) |
| Pre-procedural only | 309 (21.5) |

NB: Data are presented as a number (%) unless otherwise specified.

* Includes: implant placement (n=5), implant removal (n=5), suturing (n=1), root canal treatment (n=5), necrotic bone removal (n=4), frenectomy (n=3), washing (n=3), incision and drainage (n=3), scaling (n=3), curettage (n=2), fenestration surgery for ranula (n=2), suture removal (n=2), pulpectomy (n=2), osteoplasty (n=2), oral vitiligo excision (n=2), washing of maxillary sinus (n=1), occlusal adjustment (n=1), dental filling (n=1), caries removal (n=1), root planning (n=1), periodontal surgery (n=1), orthodontic wire (n=1), demucosation (n=1), epulis removal (n=1), crown cutting (n=1), gauze packing (n=1), cystectomy (n=1), drain removal (n=1), foreign material removal (n=1), bone transplantation (n=1), sialolith removal (n=1), gingival retraction (n=1)

** Includes cephalexin (n=1), fosfomycin (n=1), levofloxacin (n=2)

Supplementary Table 4. Details of antimicrobial prescriptions for treatment (APT) in oral surgery at outpatient dentistry centers/clinics in tertiary care hospitals (N=333)

| Characteristics | N=333 |
| --- | --- |
| Demographics |  |
| Age, year, median (range) | 63 (18-100) |
| Female sex | 151 (45.3) |
| Antimicrobial allergy | 14 (4.2) |
| Presentation |  |
| Odontalgia | 107 (32.1) |
| Mucosal swelling | 103 (30.9) |
| Mucosal discharge | 67 (20.1) |
| Facial swelling | 39 (11.7) |
| Oral swelling | 27 (8.1) |
| Abscess formation | 27 (8.1) |
| Pain on biting | 17 (5.1) |
| Trismus | 17 (5.1) |
| Fever | 3 (0.9) |
| Headache | 2 (0.6) |
| Lymphadenopathy | 2 (0.6) |
| Dentist's diagnosis related to antimicrobial prescriptions for treatment |  |
| Mandibular osteomyelitis | 83 (24.9) |
| Apical periodontitis | 37 (11.1) |
| Pericoronitis | 34 (10.2) |
| Dental abscess | 32 (9.6) |
| Facial cellulitis from odontogenic infection | 24 (7.2) |
| Acute odontogenic maxillary sinusitis | 24 (7.2) |
| Acute gingivitis | 22 (6.6) |
| Periodontitis | 14 (4.2) |
| Local infection following post tooth extraction | 11 (3.3) |
| Sialadenitis | 6 (1.8) |
| Osteonecrosis | 6 (1.8) |
| Alveolar osteitis | 5 (1.5) |
| Just-in-case* | 7 (2.1) |
| Others** | 28 (8.4) |
| Primary dental procedure |  |
| None | 238 (71.5) |
| Incision and drainage | 27 (8.1) |
| Washing | 19 (5.7) |
| Wisdom tooth or other tooth extraction | 14 (4.2) |
| Root canal treatment | 7 (2.1) |
| Teeth cleaning | 6 (1.8) |
| Others*** | 23 (6.9) |
| Antimicrobial agent |  |
| Amoxicillin | 167 (50.2) |
| Amoxicillin/clavulanate | 98 (29.4) |
| Macrolide (Clarithromycin, azithromycin) | 24 (7.2) |
| Quinolone (Levofloxacin, Sitafloxacin) | 23 (6.9) |
| Clindamycin | 15 (4.5) |
| Tetracycline (Minocycline and Doxycycline) | 5 (1.5) |
| Penicillin + clarithromycin | 1 (0.3) |

NOTE.

Data are presented as a number (%) unless otherwise specified.

* The definition of just-in-case is given in the Methods section.

** Includes: dry socket (n=4), mucositis (n=4), pericoronitis surrounding implant (n=3), pulpitis (n=3), insufficient healing post tooth extraction (n=2), salivolithiasis (n=2), oral cyst infection (n=2), necrotizing ulcerative gingivitis (n=2), hematoma (n=1), lymphangitis (n=1), chronic gingivitis (n=1), infection caused by impacted tooth (n=1), animal bite (n=1), cheilitis (n=1)

*** Includes: scaling (n=4), occlusal adjustment (n=4), curettage (n=3), suture removal(n=2), necrotic bone removal (n=2), gingival retraction (n=2), washing of maxillary sinus (n=2), dental filling (n=2), probing (n=1), implant removal (n=1).

Supplementary Figure 1. Study flow assessing the necessity and appropriateness of APP and APT


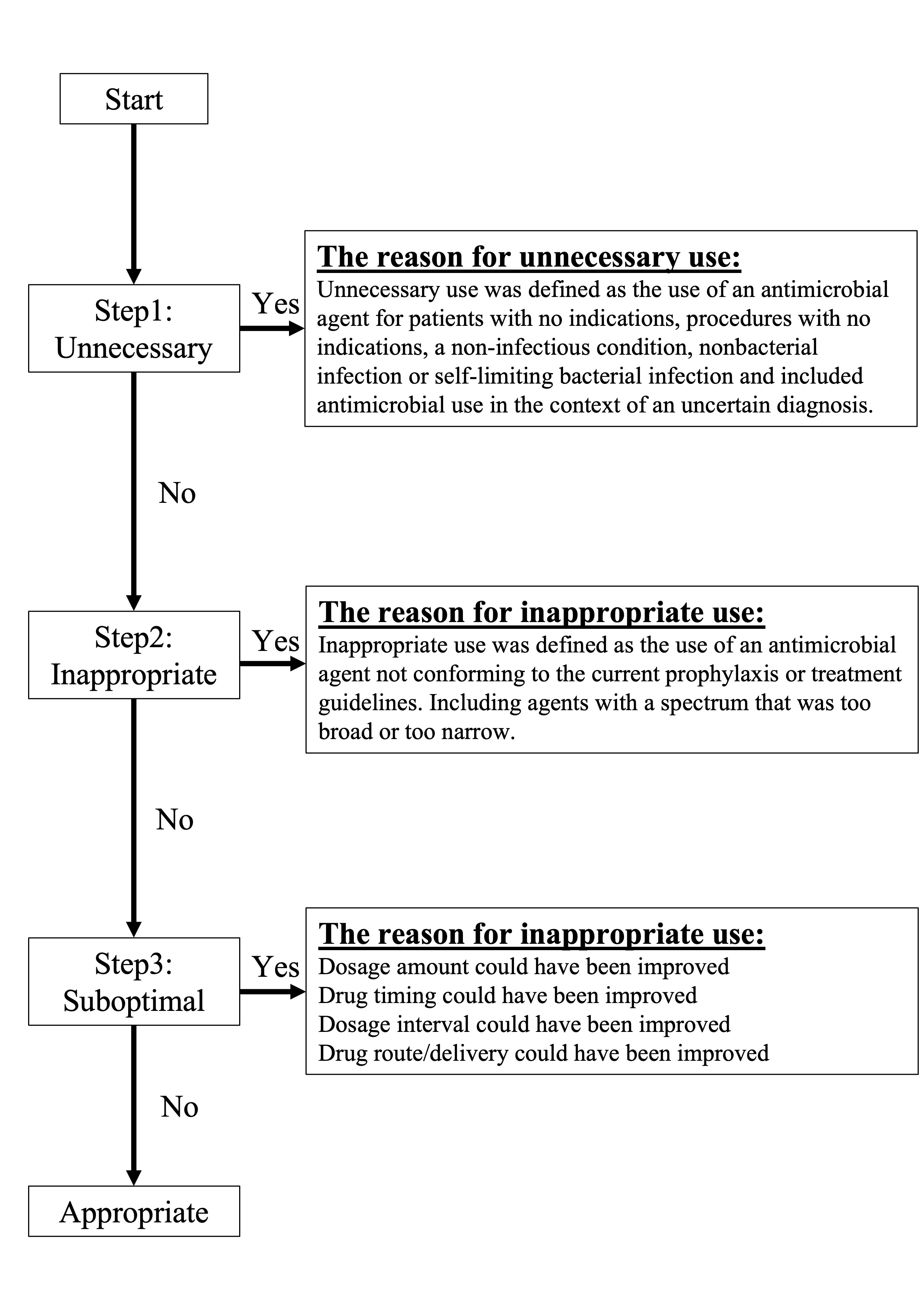


Supplementary Figure 2. Description of study population


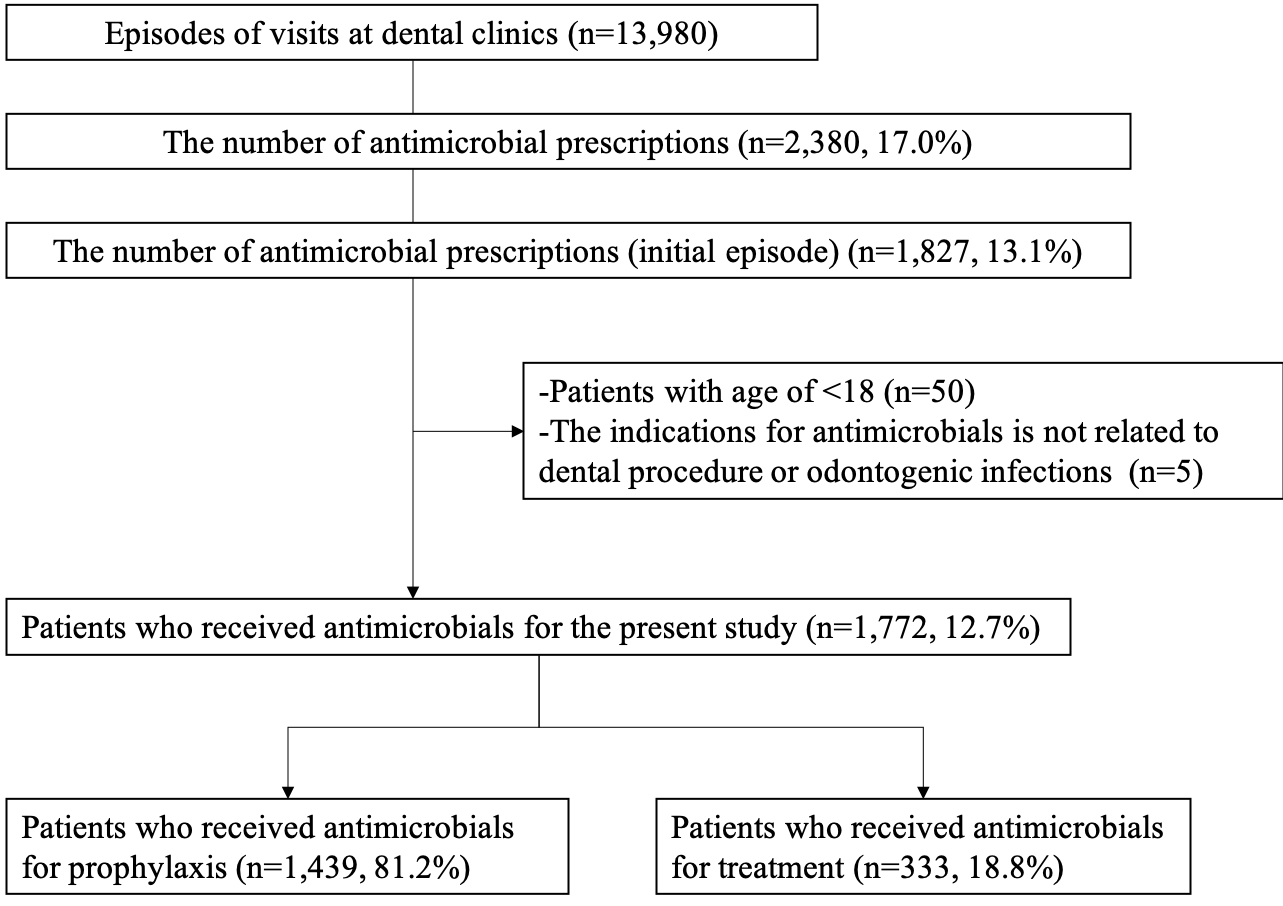


**References**

**1.** Wilson W, Taubert KA, Gewitz M, et al. Prevention of infective endocarditis: guidelines from the American Heart Association: a guideline from the American Heart Association Rheumatic Fever, Endocarditis, and Kawasaki Disease Committee, Council on Cardiovascular Disease in the Young, and the Council on Clinical Cardiology, Council on Cardiovascular Surgery and Anesthesia, and the Quality of Care and Outcomes Research Interdisciplinary Working Group. *Circulation* 2007;116:1736-1754.

**2.** Suda KJ, Henschel H, Patel U, Fitzpatrick MA, Evans CT. Use of Antibiotic Prophylaxis for Tooth Extractions, Dental Implants, and Periodontal Surgical Procedures. *Open Forum Infect Dis* 2018;5:ofx250.

**3.** Esposito M, Grusovin MG, Worthington HV. Interventions for replacing missing teeth: antibiotics at dental implant placement to prevent complications. *Cochrane Database Syst Rev* 2013:CD004152.

**4.** Lockhart PB, Loven B, Brennan MT, Fox PC. The evidence base for the efficacy of antibiotic prophylaxis in dental practice. *The Journal of the American Dental Association* 2007;138:458-474.

**5.** Sollecito TP, Abt E, Lockhart PB, et al. The use of prophylactic antibiotics prior to dental procedures in patients with prosthetic joints: Evidence-based clinical practice guideline for dental practitioners--a report of the American Dental Association Council on Scientific Affairs. *J Am Dent Assoc* 2015;146:11-16 e18.

**6.** Programme SDCE. *Scottish Dental Clinical Effectiveness Programme. Drug prescribing for dentistry: dental clinical guidance.* 3rd edition ed2011.

**7.** *Mandell, Douglas, and Bennett's Principles and Practice of Infectious Diseases.* Vol 2-Volume Set. nineth edition ed2020.

**8.** AAE Position Statement: AAE Guidance on the Use of Systemic Antibiotics in Endodontics. *J Endod* 2017;43:1409-1413.

**9.** Cope AL, Francis N, Wood F, Chestnutt IG. Systemic antibiotics for symptomatic apical periodontitis and acute apical abscess in adults. *Cochrane Database Syst Rev* 2018;9:CD010136.
